# Study on a 28-day prognostic model for ICU patients with AB infection based on machine learning

**DOI:** 10.1097/MD.0000000000046459

**Published:** 2025-12-12

**Authors:** Ying Zhang, Kai Yang, Jiepeng Huang, Jun Chen, Jiangwei Huang, Lingyan Xiao

**Affiliations:** a Department of Intensive Care Unit, The Second Hospital of Nanjing, Nanjing, China; b Department of Intensive Care Unit, The Second Hospital of Nanjing, Affiliated to Nanjing University of Chinese Medicine, Nanjing, China.

**Keywords:** *Acinetobacter baumannii*, machine learning, prognostic evaluation

## Abstract

This study aims to establish 28-day prognostic model for intensive care unit (ICU) patients with Acinetobacter baumannii (AB) infection based on machine learning. This retrospective study collected clinical data of patients admitted to the Intensive Care Medicine Department (ICU) of the Second Hospital of Nanjing from March 2021 to October 2023. The data underwent univariate and multivariate COX survival analysis, Lasso regression analysis, survival curve analysis, and model construction with SPSS and R language. The model was evaluated with area under the curves, calibration curves, and decision curve analysis curves to assess its clinical utility. Univariate COX survival analysis revealed that 11 variables: hormones, sequential organ failure assessment (SOFA), CD4 T, ThTsCD4CD8, hospital-acquired pneumonia, ventilator-associated pneumonia, pro-procalcitonin, pro-interleukin-6 (IL-6), procalcitonin, IL-6, and duration of mechanical ventilation (DurationMV) were significant risk factors for mortality in AB-infected patients (*P* < .05). In the Lasso regression analysis, lambda.1se (0.147) was selected as the optimal λ value. Variable selection was performed with the K-fold cross-validation method in the glmnet package, involving 9 influencing factors including albumin, acute physiology and chronic health evaluation II, CD4.T, SOFA, hospital-acquired pneumonia, ventilator-associated pneumonia, pro-IL6, IL-6, and DurationMV. An epwise Cox regression model was established with a global Schoenfeld test value of 0.1629. The mean square difference between survival probabilities and actual results was consistently <0.25, indicating accurate model predictions. Based on above-mentioned factors, multivariate Cox analysis showed that SOFA and DurationMV were independent risk factors for fatal outcomes, with no correlation between variables and variance inflation factor values <5. Grouping based on SOFA score showed statistically significant differences in mortality rates (*P* < .05), with higher scores associated with shorter survival times. DurationMV had greater prognostic value than the SOFA “score,” enhancing academic accuracy. Using DurationMV and SOFA variables, a survival analysis line chart model for 7, 14, and 28 days was constructed. The model was validated on 147 patients randomly divided into a training set and a validation set, achieving an area under the curve of over 80%, with calibration curves close to the diagonal line, indicating relative stability in predicting the 28-day prognosis of AB-infected patients. A 28-day prognostic model for ICU patients with AB infection based on machine learning was successfully developed and its value validated.

## 1. Introduction

Among the various bacteria in the *Acinetobacter* genus, there are significant biological and pathological differences despite belonging to the *Acinetobacter calcoaceticus-baumannii* complex.^[1]^ *Acinetobacter baumannii* (AB), a member of this genus, has been increasingly recognized in hospitals.^[2]^ AB can cause such severe conditions as bacteremia, ventilator-associated pneumonia, leading to high patient acuity and mortality rates reaching 29.8% to 36.9%.^[3]^ This poses significant challenges to clinical management and patient health. Moreover, there are notable differences in the pathogen composition of *Acinetobacter calcoaceticus-baumannii* complex bacteremia among different countries and regions.^[4]^ In terms of disease severity and prognosis, a study by Lee et al in 2013 revealed significant differences in clinical characteristics and outcomes caused by pneumonia.^[5]^ AB not only exhibits strong antibiotic resistance, limiting treatment options, but also despite relatively low pathogenicity and virulence, it is associated with exceptionally high mortality rates in infections.^[6]^ This may be due to challenges in timely administration of effective antimicrobial therapy after AB infection, exacerbated by its resistance to first-line drugs. In recent years, research on AB and its infections has made progress, deepening our understanding of this critical microbial threat and offering new treatment perspectives to clinicians.^[7]^ Advances in bacterial genomics have provided clearer insights into the unique mechanisms of drug resistance commonly found in multidrug-resistant AB, laying a theoretical foundation for developing new antimicrobial agents or optimizing the combination of existing drugs. Furthermore, improvements in rapid diagnostic tests and their combined application with antimicrobial stewardship interventions aid in faster diagnosis of AB infections and prompt initiation of effective treatment.^[8]^ However, high-quality clinical data remain scarce, making it challenging to determine the optimal treatment strategies for multidrug-resistant AB infections, leading to varying levels of patient prognostic outcomes.^[9]^ Machine learning, as a powerful data analysis tool, can extract valuable information from extensive clinical data to establish accurate predictive models. In the intensive care unit (ICU) environment, where patient conditions are complex and multifaceted, and numerous influencing factors exist, this study aims to utilize machine learning algorithms to construct a 28-day prognostic model for AB-infected patients. The goal is to provide clinicians with more precise prognostic assessments, thereby enabling the formulation of personalized treatment plans and enhancing treatment efficacy and survival rates for patients.

## 2. Materials and methods

### 2.1. Research objects

This study was approved by the Ethics Committee of The Second Hospital of Nanjing. A retrospective collection of clinical data was conducted on patients admitted to the ICU of the Second Hospital of Nanjing, China, with AB infection from March 2021 to October 2023. Hundred forty-seven cases were included, comprising 59 patients who died within 28 days and 88 survivors. This study has received approval from the Ethics Committee of the Second Hospital of Nanjing.

#### 2.1.1. Inclusion criteria

1. Diagnostic criteria for AB Infection^[10]^: Samples sent for bacterial culture were confirmed to have AB growth or predominant AB growth.
2. Patients with an ICU stay of at least 48 hours.
3. Patients aged 18 years or older.
4. For patients with multiple cultures, only data selected from the first isolation of AB were considered for patients with multiple AB-positive culture results.

#### 2.1.2. Exclusion criteria

1. Patients with AB colonization.
2. AB infection not considered a primary cause of death.
3. Patients who died or were discharged within 48 hours of ICU admission.
4. AB infection treatment duration <48 hours.
5. Patients with more than 20% missing results from multiple laboratory tests.

### 2.2. Research methods

#### 2.2.1. Investigation methodology

Clinical data of eligible patients were extracted from the hospital’s electronic medical record system according to the predefined inclusion and exclusion criteria. Data collection was performed independently by 2 researchers, and discrepancies were resolved by discussion or adjudicated by a third investigator. To ensure data quality, all variables were cross-checked for consistency and completeness. The collected data primarily included: demographic characteristics (gender, age), underlying diseases (including cardiovascular diseases, respiratory system diseases, hematological diseases, diabetes, chronic liver disease, chronic kidney disease [CKD], etc), preadmission baseline information (history of previous surgeries, history of steroid use, and history of ICU admissions), invasive procedures before and after ICU admission (continuous renal replacement therapy, mechanical ventilation, central venous catheterization), laboratory indicators (absolute neutrophil count, absolute lymphocyte count, albumin [ALB], total cholesterol, creatinine, interleukin-6 [IL-6], procalcitonin [PCT], lymphocyte subsets), site of bacterial infection, and various clinical scores within 48 hours of ICU admission (sequential organ failure assessment [SOFA], acute physiology and chronic health evaluation II [APACHE II score], Charlson comorbidity index, prognostic nutritional index [PNI]).

#### 2.2.2. Primary outcome

The primary outcome of this study is the 28-day mortality rate.

#### 2.2.3. Calculation of scores and indicators

Inflammatory indicator: Neutrophil-to-lymphocyte ratio: Calculated as neutrophil-to-lymphocyte ratio = absolute neutrophil count/absolute lymphocyte count; PNI^[11]^: Formula: PNI = serum albumin (g/L) + 5 × total lymphocyte count (×10^9^/L); APACHE II^[12]^ consists of 3 components: acute physiology score, age score, and chronic health evaluation score, with a total score ranging from 0 to 71. A higher score indicates a more severe condition and higher risk of mortality; Charlson comorbidity index^[13]^: Lists 16 common comorbid conditions, categorizing them into 4 groups based on their severity, with assigned scores of 1, 2, 3, or 6. A higher score is associated with lower survival rates; SOFA^[14]^: The SOFA score uses 6 criteria to reflect the function of organ systems (respiratory, coagulation, liver, cardiovascular, neurological, and renal systems), assigning scores of 0 to 4 for each criteria. A higher score indicates a poorer prognosis.

### 2.3. Statistical analysis

The experimental data collected were analyzed with SPSS 27.0 (International Business Machines Corporation, Armonk, NY). The Shapiro–Wilk test was employed for normality testing. Normally distributed continuous data in the experimental data were expressed as $\bar{x}\pm s$. Independent sample *t* tests were used for comparisons, while ANOVA (*F* test) was utilized for comparisons involving multiple groups. Count data were presented as frequencies or rates, with comparisons performed using *χ*^2^ test or Fisher exact test. The data underwent univariate and multivariate Cox survival analysis, Lasso regression analysis, and survival curve analysis. Models were constructed with SPSS and R programming languages. For LASSO regression, we used the glmnet package in R with a 10-fold cross-validation strategy. The model’s applicability was assessed through area under the curve (AUC) curves, calibration curves, and decision curve analysis curves. A significance level of *P* < .05 was considered statistically significant. Patients with more than 20% missing results from multiple laboratory tests were excluded. For the remaining missing data, appropriate imputation methods were applied: continuous variables (e.g., laboratory indicators) were imputed using the median of the available data, while categorical variables were imputed with the mode. This approach was adopted to reduce bias and preserve the sample size. Sensitivity analyses confirmed that the choice of imputation method did not significantly affect the stability of the prognostic model.

## 3. Results

### 3.1. Univariate cox survival analysis

Such actors as gender, age, underlying diseases, preadmission baseline information, invasive procedures before and after ICU admission, and laboratory indicators showed no statistically significant differences (*P* > .05), indicating comparability. The results of the univariate Cox survival analysis revealed that 11 variables: hormones, SOFA score, CD4 T, ThTsCD4CD8, hospital-acquired pneumonia (HAP), ventilator-associated pneumonia (VAP), pro-PCT, pro-IL6, PCT, IL-6, and duration of mechanical ventilation (DurationMV) were identified as risk factors for mortality in patients with AB infection (*P* < .05), as shown in Table 1.

**Table 1:** General data and baseline information of deceased patients.

Characteristics	Event N	HR^1^	95% CI^1^	*P*-value
Gender				.79
Female	18	–	–	
Male	41	0.93	0.53, 1.61	
Age	59	0.99	0.98, 1.01	.24
Cardiovascular				.81
0	15	–	–	
1	44	0.93	0.52, 1.67	
Respiratory	59	0.56	0.29, 1.08	.11
Diabetes	59	0.80	0.45, 1.43	.45
Liver	59	1.35	0.78, 2.36	.29
Blood system	59	1.58	0.71, 3.47	.29
Rheumatic immune system	59	1.39	0.63, 3.07	.43
CKD	59	0.97	0.52, 1.83	.93
Surgical history	59	1.12	0.67, 1.87	.68
Immunosuppressant	59	1.78	0.97, 3.24	.075
Hormones	59	1.83	1.05, 3.18	**.042**
ICU	59	1.00	0.46, 2.21	>.99
CRRT	59	1.83	0.45, 7.52	.44
Deep vein	59	1.03	0.50, 2.09	.94
ALB	59	0.95	0.90, 1.00	.067
TC	59	1.00	1.00, 1.01	.40
Cr	59	1.00	1.00, 1.00	.71
NLR	59	1.01	1.00, 1.02	.12
PNI	59	0.97	0.93, 1.01	.11
Charlson	59	1.04	0.97, 1.12	.28
SOFA	59	1.22	1.13, 1.32	**<.001**
APACHE II	59	0.99	0.95, 1.02	.41
CD3 proportions	59	1.00	0.98, 1.02	.96
CD3	59	1.00	1.00, 1.00	.14
CD4.T	59	1.00	1.00, 1.00	**.008**
CD4.CD45	59	0.98	0.96, 1.00	.051
CD8.CD45	59	1.01	1.00, 1.02	.15
CD8T	59	1.00	1.00, 1.00	.50
CD4CD8CD45	59	0.65	0.25, 1.72	.37
CD4CD8T	59	0.93	0.82, 1.04	.054
ThTsCD4CD8	59	0.81	0.65, 1.01	**.047**
CD45	59	1.00	1.00, 1.00	.073
Site	59	1.45	0.64, 3.27	.41
HAP	59	3.05	1.79, 5.20	**<.001**
VAP	59	0.30	0.18, 0.50	**<.001**
Drug allergy	59	1.04	0.95, 1.13	.45
proTmax	59	1.01	0.73, 1.40	.95
Pro-PCT	59	1.02	1.01, 1.03	**.008**
Pro-IL6	59	1.00	1.00, 1.00	**<.001**
Tmax	59	1.21	0.88, 1.66	.25
PCT	59	1.02	1.00, 1.03	**.047**
IL-6	59	1.00	1.00, 1.00	**<.001**
DurationMV	59	0.94	0.92, 0.96	**<.001**

The bold-faced values indicate that the differences were statistically significant, with *P* < 0.05.

ALB = albumin, APACHE II = acute physiology and chronic health evaluation II, CD = cluster of differentiation, CI = confidence interval, CKD = chronic kidney disease, Cr = creatinine, CRRT = continuous renal replacement therapy, HAP = hospital-acquired pneumonia, HR = hazard ratio, ICU = intensive care unit, IL-6 = interleukin-6, MV = mechanical ventilation, NLR = neutrophil-to-lymphocyte ratio, PCT = procalcitonin, PNI = prognostic nutritional index, SOFA = sequential organ failure assessment, TC = total cholesterol, Th = helper T cell, Ts = suppressor T cell, VAP = ventilator-associated pneumonia.

### 3.2. Model selection

**Figure 1.**
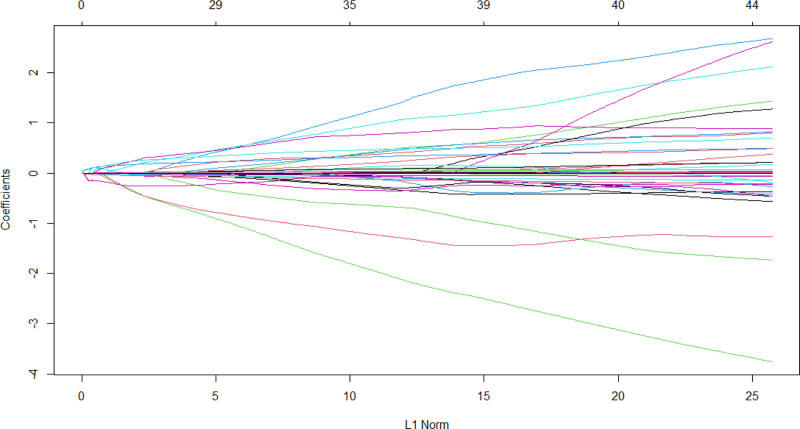
Optimal parameter (λ) selection in LASSO model.

**Figure 2.**
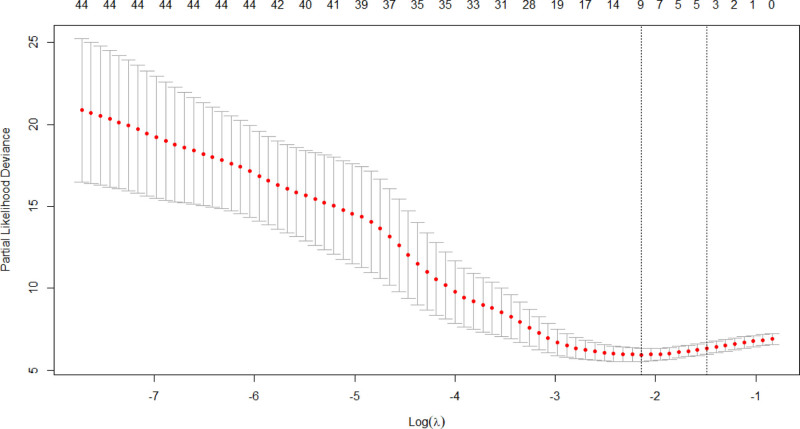
LASSO regression cross-validation results.

**Figure 3.**
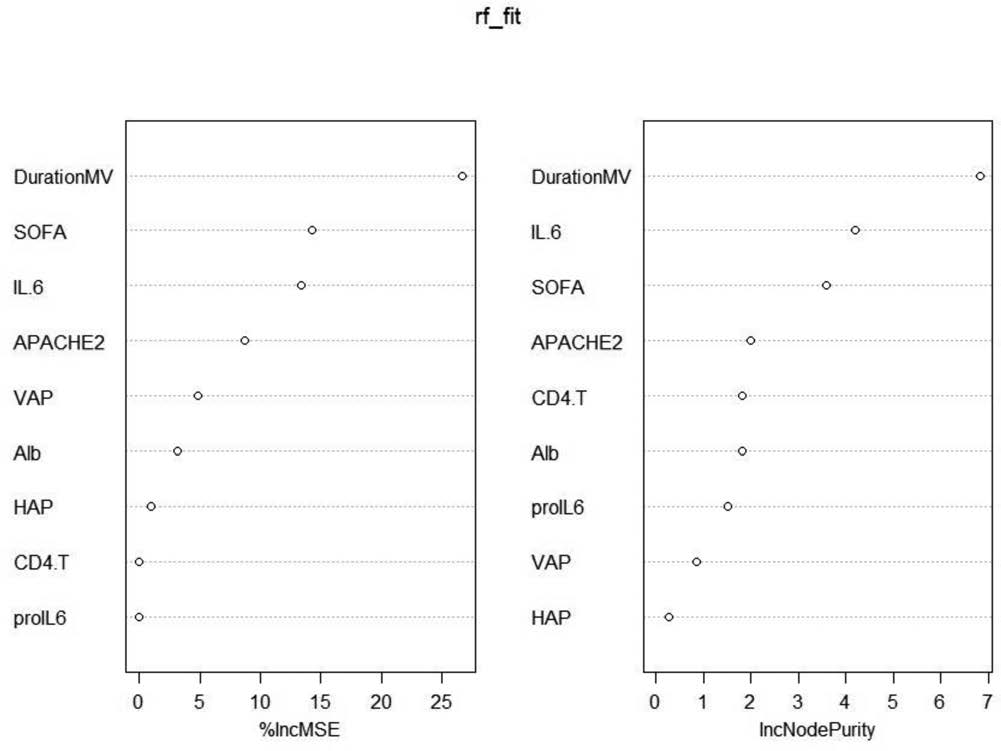
Ranking of variable importance.

For the 11 variables mentioned above, Lasso regression analysis identified lambda.1se (0.147) as the optimal λ value for model selection (see Figs. 1 and 2). Variable selection was conducted with K-fold cross-validation in the glmnet package. Factors involved in the analysis included ALB, APACHE II score, CD4 T, SOFA score, HAP, VAP, pro-IL6, IL-6, and DurationMV. These factors were ranked based on their importance (see Fig. 3).

### 3.3. Cox regression model

**Figure 4.**
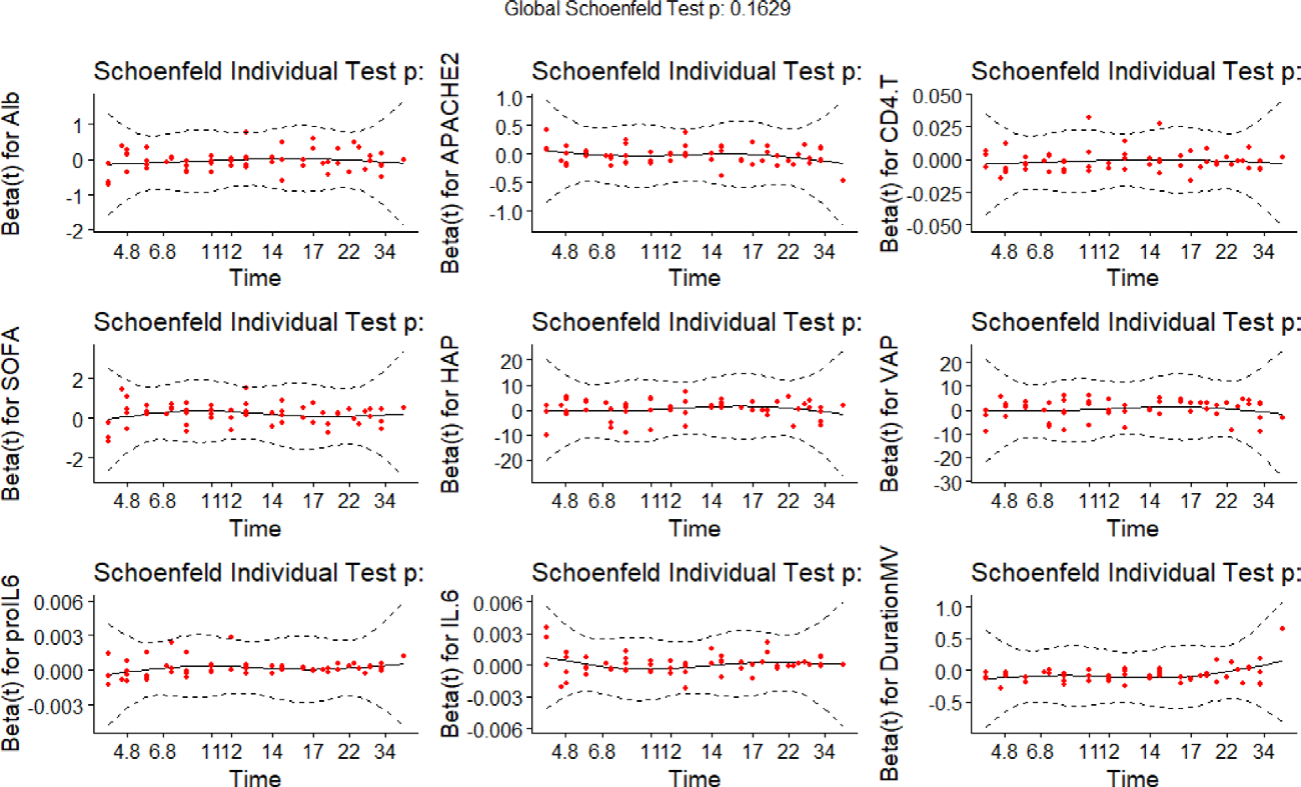
Cox regression model.

**Figure 5.**
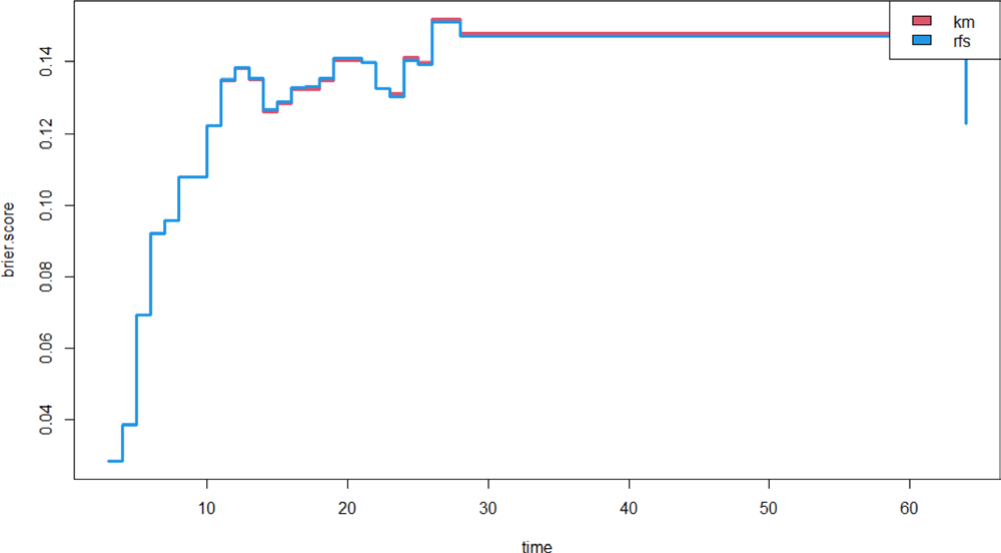
Model brier score.

An epwise Cox regression model was developed based on independent influences and the Cox proportional hazards assumption (see Fig. 4). The global Schoenfeld test yielded a value of 0.1629. The mean squared error between the survival probability curve and the actual results consistently remained <0.25, indicating that the model predictions were relatively accurate (see Fig. 5).

### 3.4. Multivariable cox analysis

In the selected model comprising variables including ALB, APACHE2, CD4.T, SOFA, HAP, VAP, pro-IL6, IL-6, and DurationMV, a multivariable Cox analysis was conducted. The results indicated that SOFA and DurationMV were independent risk factors for the fatal outcome. Furthermore, there was no correlation observed among the variables, with variance inflation factor values for each variable being <5 (see Table 2 for details).

**Table 2:** Multivariable cox survival analysis.

Characteristics	HR^1^	95% CI^1^	*P*-value	VIF^1^
ALB	0.96	0.90, 1.02	.16	1.4
APACHE2	0.98	0.94, 1.02	.25	1.4
CD4.T	1.00	1.00, 1.00	.075	1.3
SOFA	1.23	1.10, 1.37	**<.001**	1.6
HAP	1.42	0.61, 3.34	.41	2.5
VAP	1.26	0.52, 3.09	.60	3.0
Pro-IL6	1.00	1.00, 1.00	.082	1.2
IL-6	1.00	1.00, 1.00	.45	1.8
DurationMV	0.93	0.90, 0.96	**<.001**	1.6

The bold-faced values indicate that the differences were statistically significant, with *P* < 0.05.

ALB = albumin, APACHE II = acute physiology and chronic health evaluation II, CI = confidence interval, HAP = hospital-acquired pneumonia, HR = hazard ratio, IL-6 = interleukin-6, MV = mechanical ventilation, pro-IL6 = procalcitonin and interleukin-6, SOFA = sequential organ failure assessment, VAP = ventilator-associated pneumonia, VIF = variance inflation factor.

### 3.5. SOFA score stratification

**Figure 6.**
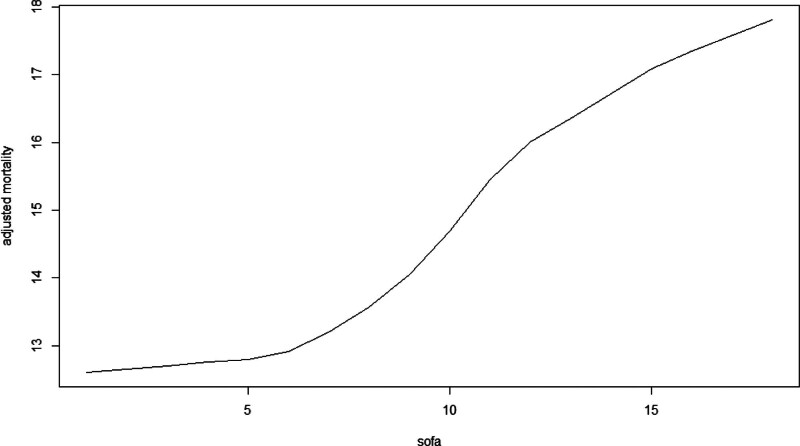
Impact of SOFA on mortality rate.

**Figure 7.**
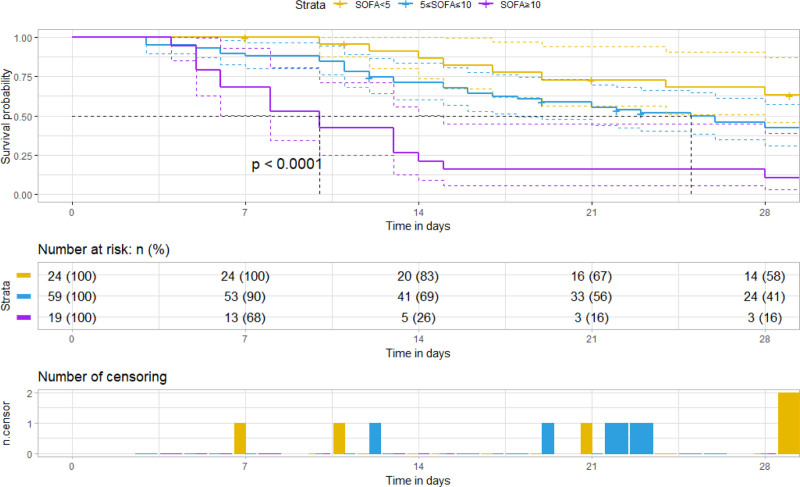
Survival curve based on SOFA score.

**Figure 8.**
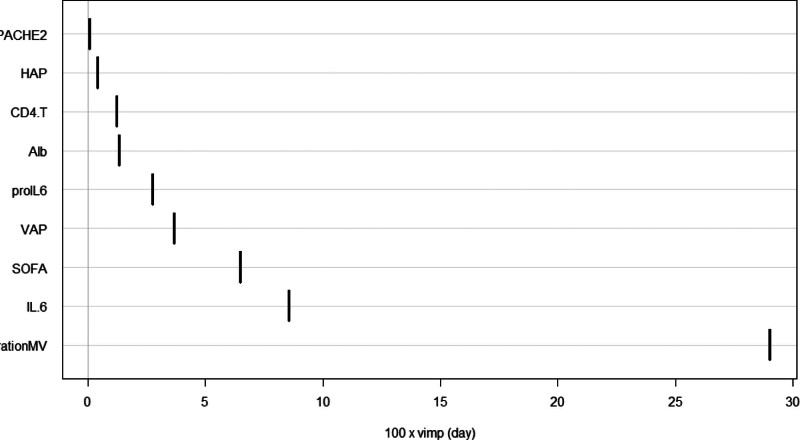
Parameter tuning.

The SOFA score was categorized into 0–5 points, 5–10 points, and 10–15 points. As the score increased, the mortality rate also rose, with statistically significant differences observed (*P* < .05) (see Figs. 6 and 7 for details). Additionally, higher scores were associated with shorter survival times (refer to Fig. 7). Notably, the importance of DurationMV exceeded that of the SOFA score (as shown in Fig. 8).

### 3.6. Nomogram model

**Figure 9.**
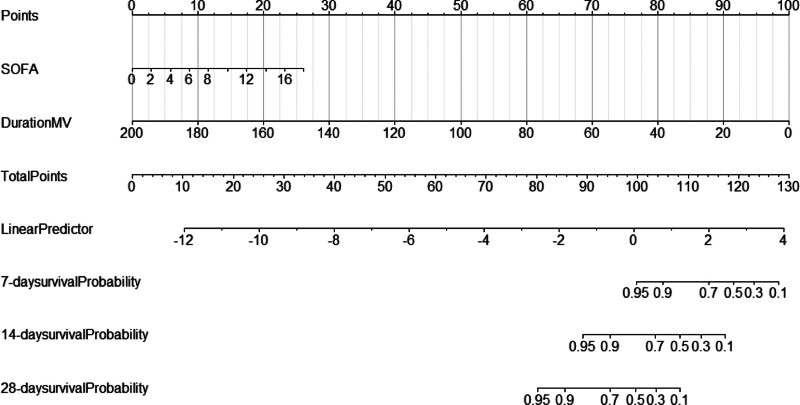
Nomogram.

Based on the results mentioned above, a nomogram model for survival analysis using the DurationMV and SOFA variables was constructed for 7, 14, and 28 days (as shown in Fig. 9).

### 3.7. Model validation

**Figure 10.**
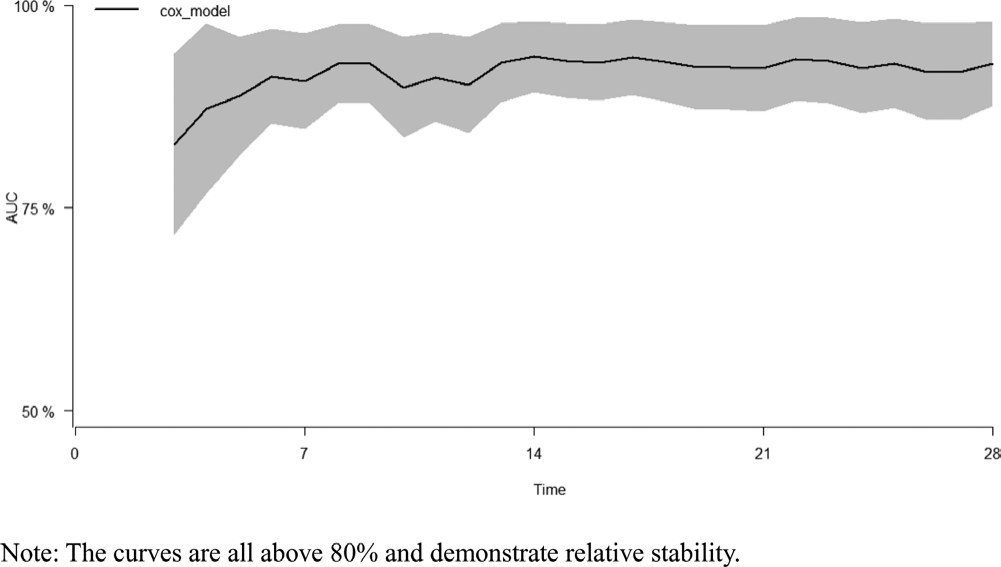
AUC of training set. The curves are all above 80% and demonstrate relative stability.

**Figure 11.**
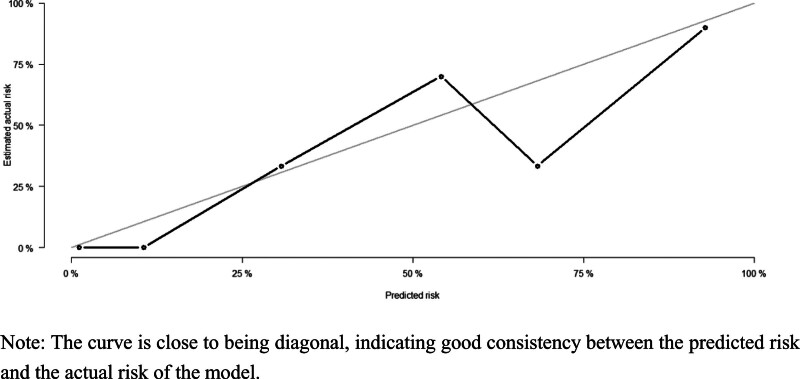
Calibration curve of training set. The curve is close to being diagonal, indicating good consistency between the predicted risk and the actual risk of the model.

**Figure 12.**
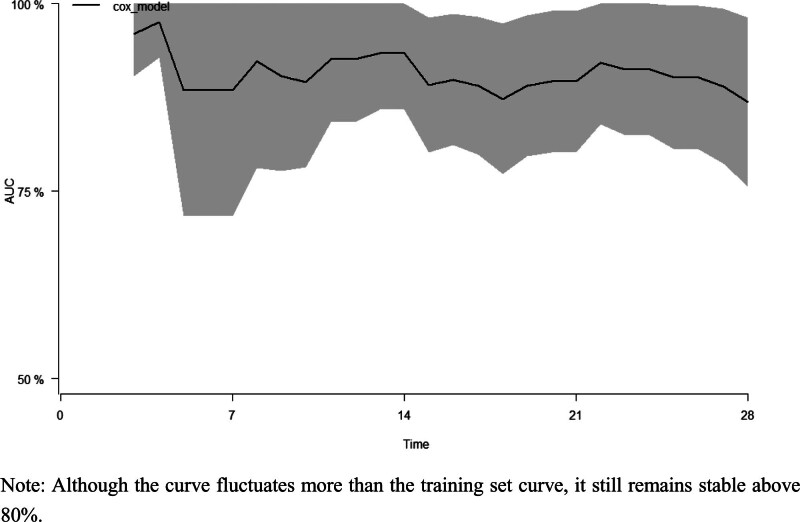
AUC of validation set. Although the curve fluctuates more than the training set curve, it still remains stable above 80%.

**Figure 13.**
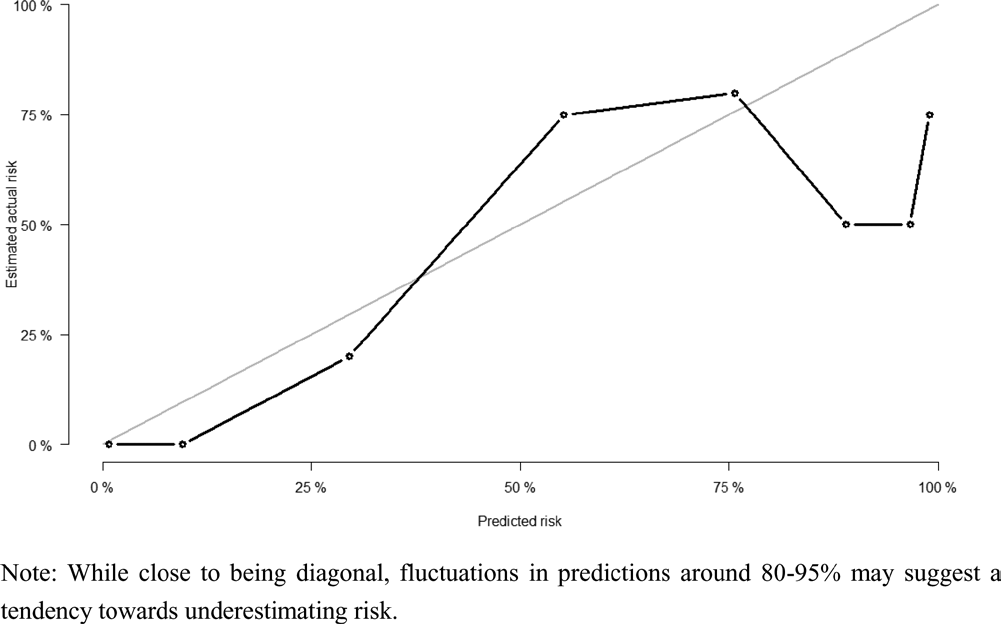
Calibration curve of validation set. While close to being diagonal, fluctuations in predictions around 80% to 95% may suggest a tendency towards underestimating risk.

Hundred forty-seven patients were randomly divided into a training set and a validation set in a 7:3 ratio. With an AUC exceeding 80%, the model demonstrated relative stability in predicting the 28-day prognosis of patients with AB infection (Figs. 10–13).

## 4. Discussion

*Acinetobacter baumannii* (AB) is a common pathogen in the ICU, and infections caused by it are associated with complex conditions and poor prognosis. Traditional prognostic assessment methods have certain limitations.^[15]^ In this study, a 28-day prognosis model for ICU patients with AB infections based on machine learning was successfully developed, providing a new assessment tool for clinical practice.

Traditional prognostic assessment methods heavily rely on clinicians’ clinical experience and some simple scoring systems, which have inherent subjectivity and limitations. In this study, univariate Cox survival analysis identified 11 mortality risk factors, with 9 influencing factors selected through Lasso regression. Multivariate Cox analysis revealed that SOFA and DurationMV are independent risk factors. Previous research by Zhao et al^[16]^ demonstrated that DurationMV is an independent factor affecting mortality rates, which aligns closely with the findings of this study, further validating the results. Based on this, prognostic line graph models for 7, 14, and 28 days were constructed, with model validation showing AUC exceeding 80% and calibration curves approaching linearity, indicating relative stability in the 28-day prognosis predictions. Earlier study by Li et al^[17]^ also constructed a mortality prediction model for patients with AB bloodstream infections; however, it did not incorporate machine learning models. By integrating a machine learning model, this study can provide a more comprehensive and accurate evaluation of patient prognosis.

In clinical practices in the ICU, doctors face pressure to make accurate treatment decisions within a short timeframe. Previous research by Lortholary et al^[18]^ analyzed prognostic risk factors for ICU patients with AB infections; however, without establishing a comprehensive predictive model, their applicability is limited. The prognostic model developed in this study can serve as an effective decision support tool. For instance, when encountering patients with AB infections, doctors can input such relevant indicators as SOFA score and DurationMV into the model to quickly obtain a 28-day prognosis prediction for the patient. For patients predicted to have a poor prognosis, doctors can consider more aggressive treatment measures earlier, such as adjusting antibiotic regimens or enhancing immunotherapy. Conversely, for patients with a relatively good prognosis, overtreatment can be avoided, reducing wastage of medical resources and unnecessary patient suffering.^[19]^ Furthermore, the model aids in stratified management of ICU patients with AB infections. Based on the model’s predictions, patients can be categorized into high, medium, and low-risk groups. For high-risk patients, closer monitoring and higher levels of care can be provided to promptly detect changes in their condition and administer appropriate treatment. Medium-risk patients may have their monitoring frequency and treatment plans adjusted to improve healthcare resource utilization efficiency while maintaining treatment effectiveness. Low-risk patients can receive relatively standard treatment and care, minimizing unnecessary medical interventions.^[20,21]^ This tiered management approach enhances overall ICU management efficiency and improves patient treatment outcomes. Moreover, in situations of limited medical resources, rational resource allocation is crucial. The prognostic model from this study can guide resource allocation decisions. By prioritizing the allocation of more medical resources to patients with poorer prognoses and higher treatment complexities, the success rates of treating these patients can be increased. Conversely, for patients with relatively good prognoses, resource input can be appropriately reduced to avoid wastage.^[22]^ Lastly, the model can also be utilized for disease monitoring and research purposes. By analyzing prognosis data from a large number of patients, trends in AB infection prevalence and changes in risk factors can be understood. Additionally, the model’s establishment provides a foundation for further research into the pathogenesis and treatment methods of AB infections. For instance, researchers can use the model to identify groups of patients with poorer prognoses for in-depth studies on etiology and pathophysiology, seeking new treatment targets and methods. Furthermore, by comparing prognosis data from patients in different time periods and regions, the effectiveness of various treatment measures can be evaluated, providing a basis for the development and updating of clinical guidelines.

The 28-day prognosis model for ICU patients with AB infections developed in this study holds significant importance in various aspects of medical practice, particularly in personalized medicine, multidisciplinary collaboration, and healthcare quality improvement. In the realm of personalized medicine, the individual conditions and health statuses of each AB infection patient vary, making traditional “one-size-fits-all” treatment approaches inadequate for meeting individual needs. The prognosis model, however, can conduct precise personalized prognostic assessments based on the individual characteristics of patients and provide treatment recommendations accordingly. Regarding the advancement of healthcare quality improvement, the establishment and application of the model drive hospitals to standardize and normalize the treatment processes for ICU patients with AB infections. By comparing the model’s predicted outcomes with actual treatment results, issues in clinical care can be identified, leading to the formulation of improvement measures.

While the prognostic model developed in this study holds certain application value, it also has some limitations. For instance, we acknowledge that retrospective studies are inherently subject to selection bias, information bias, and confounding factors that cannot always be fully controlled. Although we used strict inclusion/exclusion criteria and data quality control measures, residual bias may still exist. Since all patients were recruited from a single tertiary hospital, the study population may not fully represent patients in other institutions or regions. Differences in ICU management practices, antibiotic stewardship, and patient characteristics could limit the external validity of our findings. Future studies should aim to validate this model in larger, multicenter, prospective cohorts to enhance generalizability. Additionally, incorporating more advanced technologies like genomics, proteomics, and others could further refine the model’s construction, providing more precise support for the treatment and management of ICU patients with AB infections.

## 5. Conclusion

In conclusion, the 28-day prognosis model for ICU patients with AB infections based on machine learning holds significant application value and clinical significance. It provides robust support for clinical decision-making, patient management, healthcare resource allocation, and disease research, aiding in enhancing the treatment outcomes and improving the prognosis of AB infection patients. With ongoing research advancements and technological developments, this model is poised to play a greater role in clinical practices.

## Author contributions

**Conceptualization:** Ying Zhang, Kai Yang, Lingyan Xiao.

**Data curation:** Ying Zhang, Kai Yang, Jun Chen, Jiangwei Huang, Lingyan Xiao.

**Formal analysis:** Ying Zhang, Kai Yang, Lingyan Xiao.

**Investigation:** Ying Zhang, Jiepeng Huang, Jiangwei Huang, Lingyan Xiao.

**Methodology:** Ying Zhang, Jiepeng Huang, Jiangwei Huang, Lingyan Xiao.

**Supervision:** Ying Zhang, Kai Yang, Jiepeng Huang, Jun Chen, Jiangwei Huang.

**Validation:** Ying Zhang, Kai Yang, Jiepeng Huang, Jun Chen, Jiangwei Huang.

**Visualization:** Ying Zhang, Kai Yang, Jiepeng Huang, Jun Chen.

**Writing – original draft:** Ying Zhang, Lingyan Xiao.

**Writing – review & editing:** Ying Zhang, Lingyan Xiao.
